# Supplementary figures and images for: Spontaneous Confinement of mRNA Molecules at Biomolecular Condensate Boundaries
Source: Cells. 2023 Sep 11;12(18):2250. doi: 10.3390/cells12182250 (PMC10526803; doi:10.3390/cells12182250)

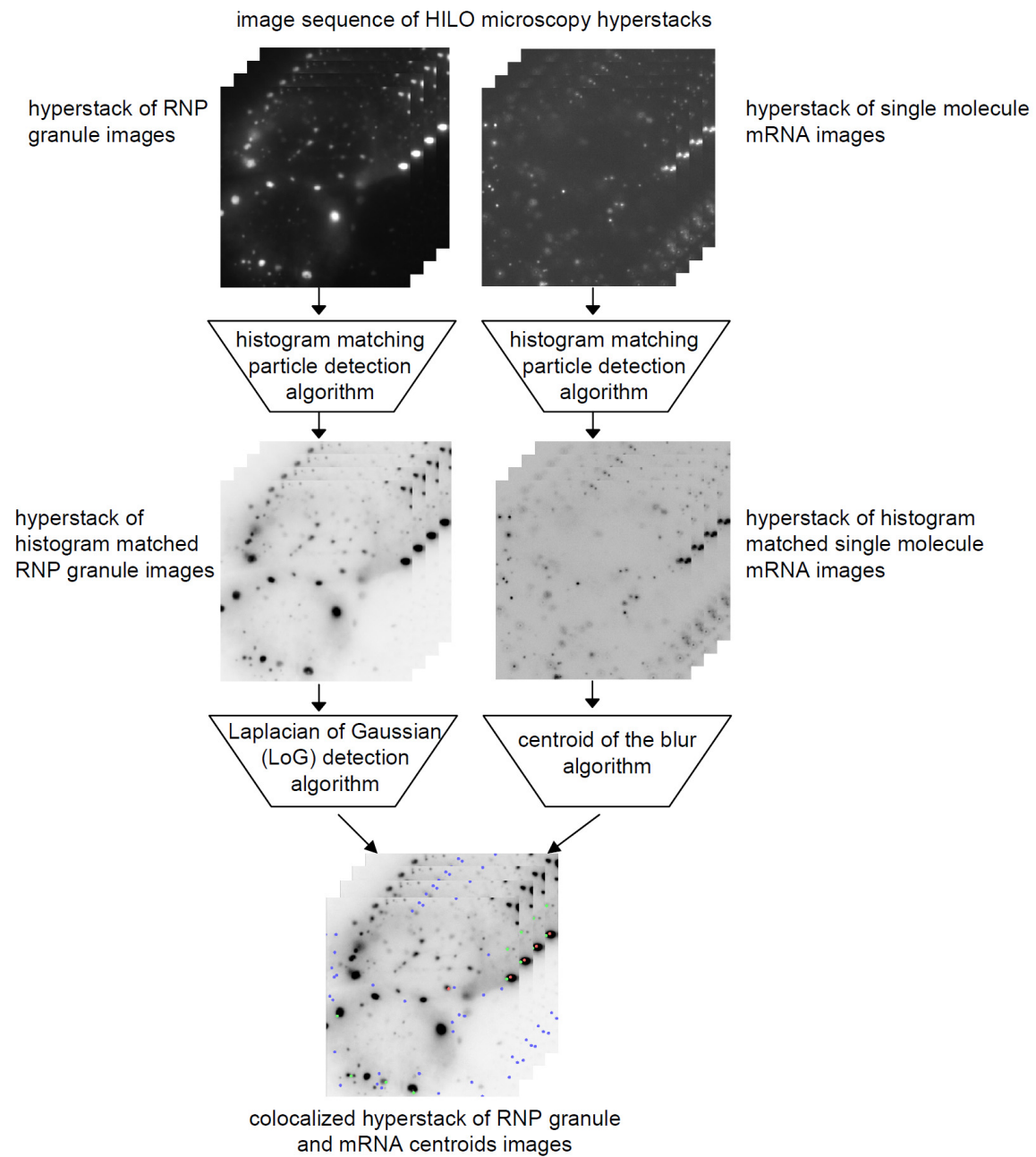

**Figure S1.** Workflow diagram outlining the image processing and colocalization algorithm.

Supplement: Supplementary file 1 [file cells-12-02250-s001.zip › cells-2590385-supplementary revise .pdf]
